# Revisiting the Phylogeny of the Animal Formins: Two New Subtypes, Relationships with Multiple Wing Hairs Proteins, and a Lost Human Formin

**DOI:** 10.1371/journal.pone.0164067

**Published:** 2016-10-03

**Authors:** David Pruyne

**Affiliations:** Department of Cell and Developmental Biology, State University of New York Upstate Medical University, Syracuse, NY, United States of America; Centre for Research and Technology-Hellas, GREECE

## Abstract

Formins are a widespread family of eukaryotic cytoskeleton-organizing proteins. Many species encode multiple formin isoforms, and for animals, much of this reflects the presence of multiple conserved subtypes. Earlier phylogenetic analyses identified seven major formin subtypes in animals (DAAM, DIAPH, FHOD, FMN, FMNL, INF, and GRID2IP/delphilin), but left a handful of formins, particularly from nematodes, unassigned. In this new analysis drawing from genomic data from a wider range of taxa, nine formin subtypes are identified that encompass all the animal formins analyzed here. Included in this analysis are Multiple Wing Hairs proteins (MWH), which bear homology to formin N-terminal domains. Originally identified in *Drosophila melanogaster* and other arthropods, MWH-related proteins are also identified here in some nematodes (including *Caenorhabditis elegans*), and are shown to be related to a novel MWH-related formin (MWHF) subtype. One surprising result of this work is the discovery that a family of pleckstrin homology domain-containing formins (PHCFs) is represented in many vertebrates, but is strikingly absent from placental mammals. Consistent with a relatively recent loss of this formin, the human genome retains fragments of a defunct homologous formin gene.

## Introduction

Formins are best known as regulators of actin filament dynamics. These proteins are critical for the assembly of a variety of actin-based cellular structures, including but not limited to cytokinetic contractile rings, stress fibers, and cables that mediate actin-dependent intracellular transport (reviewed in [1–3]). Of clinical significance, mutations in human formin genes have been linked to nonsyndromic deafness [4], focal segmental glomerulosclerosis affecting the kidney [5], the neuropathology Charcot-Marie-Tooth disease [6], hypertrophic and dilated cardiomyopathies [7, 8], microcephaly [9], and nonsyndromic intellectual disability [10].

The defining feature of formin proteins is a ~ 350 amino acid residue actin-binding formin homology-2 (FH2) domain that is often paired with an upstream proline-rich formin homology-1 (FH1) domain [2]. Many formins encode additional actin-binding sites in the form of one or two C-terminal Wiskott-Aldrich syndrome protein homology-2 (WH2)-like motifs [11–14]. These domains, often working in conjunction with additional actin-binding proteins, can exert a variety of effects on actin filaments, including promoting actin filament nucleation, severing, elongation, and bundling [11–15].

The Diaphanous-related formins (Drfs) are a subset of these proteins that have additional conserved regions N-terminal to their FH1 domain, including a RhoGTPase-binding domain (G-domain) followed by a diaphanous inhibitory domain (DID), and a dimerization domain (DD). The DD promotes homodimerization, while the DID of many Drfs binds to a C-terminal WH2-like motif (called in this case a diaphanous autoregulatory domain, or DAD) to hold the formin in an autoinhibited state [2, 16]. Relief of Drf autoinhibition often includes binding of a RhoGTPase to the G-domain and DID in a manner that helps disrupt the DID/DAD interaction [3]. The property of autoinhibition has sometimes been used as a defining criterion for whether or not a formin is a Drf, but for the purposes of this work, Drf will simply refer to a formin with an N-terminal domain organization of G-DID-DD.

It should also be noted that the designation "Drf" is something of a misnomer, as a distinct diaphanous (also called DIAPH) subtype of formins represents just one of many Drf-type formins. In fact, the majority of metaozoan formins are Drfs, but a significant minority of formins are non-Drfs that diverge from this domain organization. In such non-Drfs, one or more conserved N-terminal domains are absent, often to be replaced by other folds, such as structurally distinct GTPase-binding domains, or postsynaptic density protein 95/ Drosophila disc large tumor suppressor 1/zonula occludens-1 protein (PDZ) domains [16]. These alternative domains presumably exert their own unique effects in regulating the subcellular localization or activity of non-Drfs.

Based on phylogenetic analyses of FH2 domains, metazoan Drf and non-Drf formins can be further subdivided into seven subtypes that are conserved across multiple phyla [17, 18]. Using a naming convention based on a representative human gene, these subtypes are denoted here as: the Drf-type diaphanous proteins (DIAPHs), formin-like proteins (FMNLs), and disheveled-associated activator of morphogenesis proteins (DAAMs); the non-Drf-type canonical formins (FMNs,), formin homology domain-containing proteins (FHODs), and glutamate receptor ionotropic delta 2-interacting proteins/delphilins (GRID2IPs); and finally, the N-terminally truncated Drf-like inverted formins (INFs). However, a number of animal formins and formin-like proteins have not fit neatly into these subtypes in previous phylogenetic analyses. One example is a non-Drf formin identified in the cnidarian *Nematostella vectensis* that was unique among known animal formins for the presence of N- and C-terminal pleckstrin homology (PH) domains [19]. Nematode FH2 domain-containing proteins provide additional examples. Most notable among these is FOZI-1 of *Caenorhabditis elegans*, whose only formin homology is a highly divergent FH2 domain that has resisted previous assignment to a conserved formin subtype [18–22]. Finally, Multiple Wing Hairs (MWH) of *Drosophila melanogaster* shares incomplete similarity to formins, with sequence homologous to the N-terminal domains of Drfs, but lacking FH1, FH2, or other conserved C-terminal formin homology [23, 24]. As with FOZI-1, the formin-related regions of MWH have defied categorization to a particular formin subtype [25].

The availability of more complete genomic data from a wider range of taxa provided an opportunity to revisit the phylogeny of this important family of cytoskeleton-organizing proteins. As presented here, a broader sampling of formins helped reveal two new groups that were not previously recognized as being broadly distributed across metazoans, and tied the origins of MWH- and FOZI-1-related proteins to specific formin subtypes. Additionally, evidence is presented of an ancestral formin from one of these novel families that was lost recently from the lineage containing the placental mammals.

## Materials and Methods

### Identification of FH2 domain-containing proteins and MWH homologs

Formins were identified through searches for FH2 domains in species for which at least a draft genomic sequence was available. Specifically, protein databases and translated nucleotide databases were searched using the Basic Local Alignment Search Tool (BLAST) [26]. Publically available databases were accessed through: the National Center for Biotechnology Information (NCBI) website (blast.ncbi.nlm.nih.gov/blast.cgi) for *Homo sapiens*, *Mus musculus*, *Monodelphis domestica*, *Gallus gallus*, *Danio rerio*, *Ciona intestinalis*, *Strongylocentrotus purpuratus*, *Drosophila melanogaster*, *Limulus polyphemus*, *Crassostrea gigas*, *Lottia gigantea*, *Helobdella robusta*, and *Amphimedon queenslandica*; the Ensembl Genomes website (www.ensemblgenomes.org) [27] for *Daphnia pulex*, *Capitella teleta*, *Nematostella vectensis*, *Mnemiopsis leidyi*, *Trichoplax adhaerens*, and *Amphimedon queenslandica*; the WormBase website (wormbase.org; version WS252) [28] for *Caenorhabditis elegans*; and the WormBase ParaSite (parasite.wormbase.org; version WBPS6) [29] for *Ascaris suum*, *Strongyloides ratti*, *Romanomermis culicivorax*, *Trichuris suis*, *Clonorchis sinensis*, and *Echinococcus granulosus*. Searches were conducted using standard search parameters. To ensure all FH2 domains were detected, each species was subject to search queries based on FH2 domains from the *M*. *musculus* formins DAAM1, DIAPH1, FMN2, GRID2IP, FHDC1 (an INF-subtype formin), INF2, FMNL1, and FHOD1, the *S*. *purpuratus* formin LOC100890634 (a pleckstrin homology domain-containing formin), and the *C*. *elegans* FOZI-1 (a divergent FMNL-subtype protein). Searches were not conducted using a representative of the novel MWH-related formin (MWHF) subtype, as these proteins were not initially recognized as a distinct subtype. However, the similarity between MWHF and FMNL proteins makes it unlikely that any formins were missed due to this omission. All identified formins are listed by species in S1 Table.

The same species were searched for MWH homologs by querying for homology to predicted DID and DD sequences of *D*. *melanogaster* MWH. Positive hits of high significance, as occurred with other arthropods, were accepted with no further confirmation. Positive hits of marginal significance that were found for certain nematodes were further tested by using the nematode sequences as the basis for additional queries. Proteins for which reversed queries identified other MWH proteins were considered to be MWH homologs. All identified MWH proteins are also listed by species in S1 Table.

Subsequent domain and multiple sequence alignment analyses suggested some predicted FH2 domain sequences were incomplete, while others were not coupled with expected additional regions of formin homology. Such cases were almost always based on gene predictions, rather than isolated cDNA sequences. Working on the assumption that these reflected errors in annotation, the genomic sequences were examined for additional formin-coding sequence, as follows. In cases of presumed internal gaps of the FH2 domain, predicted intron sequences that occupied gap regions were translated in three frames and searched for FH2 similarity, and any identified unambiguous FH2-coding sequence was restored. Formins for which this was done are labeled "inferred" in S1 Table. In cases of presumed gaps at one end of the predicted FH2-coding sequence, or in cases where sequence for additional expected regions of homology were absent (e.g. absent FH1- or DID-coding sequences), adjacent annotated genes and intervening sequences were searched for formin homology. Again, unambiguous formin-homologous sequences were restored, and such formins are also labeled "inferred" in S1 Table. In cases where two adjacent genes appeared to encode pieces of the same formin, both genes are noted in S1 Table and in all phylogenetic trees. In cases where presumed gaps could not be restored in FH2 domain sequences, such as in cases of unsequenced genomic intervals, those formins are labeled "partial FH2" in S1 Table and indicated with an asterisk in all phylogenetic trees. The designation "partial" is used in S1 Table to denote formins for which presumably absent non-FH2-regions of formin homology could not be identified due to incomplete sequence data.

### Domain analysis

Formin and MWH amino acid sequences were subject to Conserved Domain Searches (CDS) [30] by comparison against the NCBI Conserved Domain Database superset [31]. Standard search parameters were used, with the exception of an Expect value threshold of 1.0, providing a less stringent search more likely to identify poorly conserved domains. CDS results were used to define the boundaries of FH2, PDZ, and Harmonin N-terminus-like domains, and as preliminary indicators of additional structural sequences.

DAD and other WH2-like motifs were only rarely identified by CDS, and were missed in many cases where DAD and WH2 motifs were shown to exist in previous studies. Similarly, attempts to identify these motifs using the Eukaryotic Linear Motif resource [32] were also almost always unsuccessful. Instead, DAD and WH2 motifs were identified manually after alignment to related formins for which those motifs had been previously noted, including *M*. *musculus* DIAPH1, DIAPH2, DIAPH3, DAAM1, DAAM2, FHOD1, FHOD3, FMNL1, FMNL2, FMNL3, and INF2, *D*. *melanogaster* DIA, DAAM, FRL, and FHOS, and *C*. *elegans* FHOD-1 and FRL-1 [11, 12, 14, 17]. No novel DAD/WH2-like sequences were identified in any FMN, GRID2IP, MWHF, or PHCF homolog. FH1 domains were also identified manually as any segments of two or more adjacent prolines, plus all the sequence between these, that were positioned N-terminal to the FH2 domain.

For positive identification of other structural domains, formin sequences N-terminal to FH1 domains, formin sequences C-terminal to FH2 domains, and entire MWH sequences, were submitted to the Protein HomologY Recognition Engine 2 (PHYRE^2^) website (www.sbg.bio.ic.ac.uk/phyre2/html/page.cgi?id=index) [33]. Only domain structure predictions accompanied by a confidence of homology ≥ 95% were considered likely. The single exception to this was acceptance of an 86.6% level of confidence in homology for predicted conserved N-terminal zinc fingers of the *S*. *ratti* FOZI-1-like protein SRAE_2000156800.

### Multiple sequence alignments

FH2 domain amino acid sequences were aligned by the ClustalW method [34] using MegAlign (version 13.0.0) of the Lasergene software suite (DNASTAR, Madison, WI), with the default settings (Gap penalty 10, gap length penalty 0.2) and a Gonnet Series matrix. To obtain a multiple sequence alignment of DIDs, a preliminary alignment of all sequences preceding the FH1 domain was generated using ClustalW in MegAlign. These preliminary alignments were then trimmed to exclude poorly aligned sequences flanking the conserved DID core. Alignments of combined DID and DD (DID-DD) were created in a similar manner. Initial alignments were manually corrected for gross mistakes that were typically the result of large gaps or long insertions in individual input sequences. Such manual corrections were done using groups of highly conserved amino acid residues as landmarks. Ten residue sequences of core DAD and WH2-like motifs were aligned with no manual adjustments. All sequence alignments are presented in an interleaved format in S1 Text.

### Estimation of phylogenies

Evolutionary histories for aligned FH2 domain, DID-DD, or DID sequences were inferred using the Maximum Likelihood (ML) method in the MEGA6 program [35]. Out of 48 models of amino acid substitutions, the LG model [36] + G (using a discrete Gamma distribution with 5 categories to model evolutionary rate differences among sites) [37] was selected for producing the lowest Bayesian Information Criterion score [38] when tested using MEGA6. Initial trees were obtained by applying the Neighbor-Joining (NJ) method [39] to a matrix of pairwise distances estimated using a Jones-Taylor-Thorton model. Where possible, trees were also estimated using the NJ method with evolutionary distances computed using the Poisson correction method [40], again in MEGA6. All trees were tested by bootstrap analysis, with 100 or 250 replicates. For alignments that contained only complete FH2 domain sequences, positions for which any sequence contained a gap were excluded from consideration. For all other alignments, positions were excluded only when they were unoccupied in a large enough percentage of sequences such that positions occupied in full-length sequences were not omitted. Unrooted trees were generated using MEGA6, with branch lengths proportional to the number of substitutions per site. Evolutionary histories were also estimated for DAD/WH2-like motifs. Resulting phylogenetic trees showed little correlation with those estimated for FH2, DID-DD, or DD, likely due to the short length and poor conservation of the DAD/WH2-like motifs. Thus, these were considered unreliable and are not presented here.

### Synteny analysis

Gene order in chromosomal neighborhoods of various vertebrate species were examined using the Genomicus v84.01 website (www.genomicus.biologie.ens.fr/genomicus-84.01/cgi-bin/search.pl) [41]. Depictions of gene positions were generated from two PhyloViews that used the *CBLN4* and *MC3R* genes, respectively, of the opossum *M*. *domestica* as references in comparison to all available bilaterian genomes.

## Results

### Nine metazoan formin subtypes

To analyze the phylogeny of metazoan formins, BLAST searches [26] were used to identify FH2 domain-containing sequences. In order to sample the animal kingdom broadly, species were selected from phyla representing all major parts of the metazoan family tree (see S1 Table for a listing of all identified formins by species). This included representatives from three phyla commonly considered basal branches of the tree, porifera, placozoa, and ctenophora, as well as a representative of cnidaria [42–44]. Representatives were also selected from phyla belonging to each of the bilaterian superphyla: mollusca, annelida, and platyhelminthes for lophotrochozoa; arthropoda and nematoda for ecdysozoa; and echinodermata and chordata for deuterostomia. For an initial analysis, one representative from each of ten of these phyla was chosen for having nearly complete FH2 domain sequences for all their known formins. Additionally, a set of complete and incomplete FH2 domain sequences was included from the sole representative of the phylum cnidaria.

**Fig 1:**
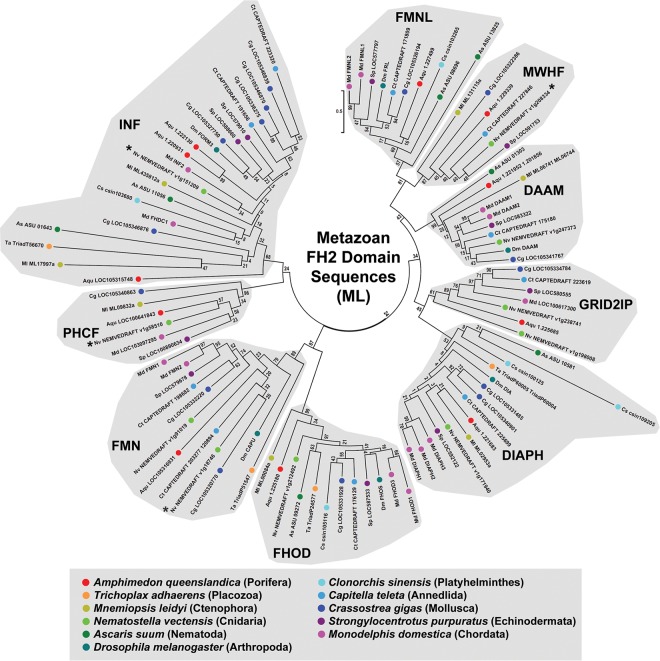
Nine evolutionarily conserved metazoan FH2 domain subtypes. The evolutionary history for 100 FH2 domain amino acid sequences from representatives of eleven metazoan phyla was inferred by the ML method for 343 amino acid positions occupied in ≥ 95% of sequences. All bootstrap values are indicated, and the scale bar indicates the number of substitutions per site for branch lengths. Nine groups populated by formins from multiple species clustered behind nodes with bootstrap values ≥ 50, suggesting the presence of nine evolutionarily conserved subtypes. Seven of these conformed to the previously recognized DAAM, DIAPH, FHOD, FMN, FMNL, INF and GRID2IP subtypes, while two others, designated MWHF and PHCF, were novel. Asterisks (*) indicate formins for which a partial FH2 domain sequence was used for this analysis.

These FH2 domain amino acid sequences were aligned (S1 Text, Alignment 1), and ML and NJ phylogenetic trees were estimated (Fig 1 and S1 Fig). Within the trees, groupings of formins were considered to represent evolutionarily conserved subtypes if they included formins from multiple animal phyla, and if they were segregated from the rest of the tree by a node recovered in ≥ 50% bootstrap replicates in both trees. Nine formin subtypes were defined based on these criteria, with all the analyzed formins falling into one of these nine. As a further test for the robustness of the nine subtypes, complete and partial FH2 domain sequences from fourteen additional bilaterian animal species were collected and aligned (S1 Text, Alignment 2), and a ML phylogenetic tree including these was estimated (S2 Fig). With the exception of a handful of divergent nematode proteins (discussed later), the organization of formins into the same nine subtypes was preserved. Seven previously described subtypes (DAAM, DIAPH, FHOD, FMN, FMNL, INF, and GRID2IP) were recovered, and two additional groups were revealed. For reasons explained below, these two new subtypes are designated here as PHCF and MWHF proteins.

Strongly supported nodes also appeared within putative subtypes, but these nodes rarely separated multiple formins from a single species, as would be expected if they defined additional formin subtypes. The only exception found in both the ML and NJ trees was a node within the FMN subtype that divided multiple mollusk, annelid, and cnidarian FMN homologs into two groups, potentially indicating a further conserved subdivision of the FMN subtype. However, the relationship of formins from other phyla to these putative subtypes was not clear, and this was not investigated further.

### A family of PH domain-containing formins widely distributed across metazoa

**Fig 2:**
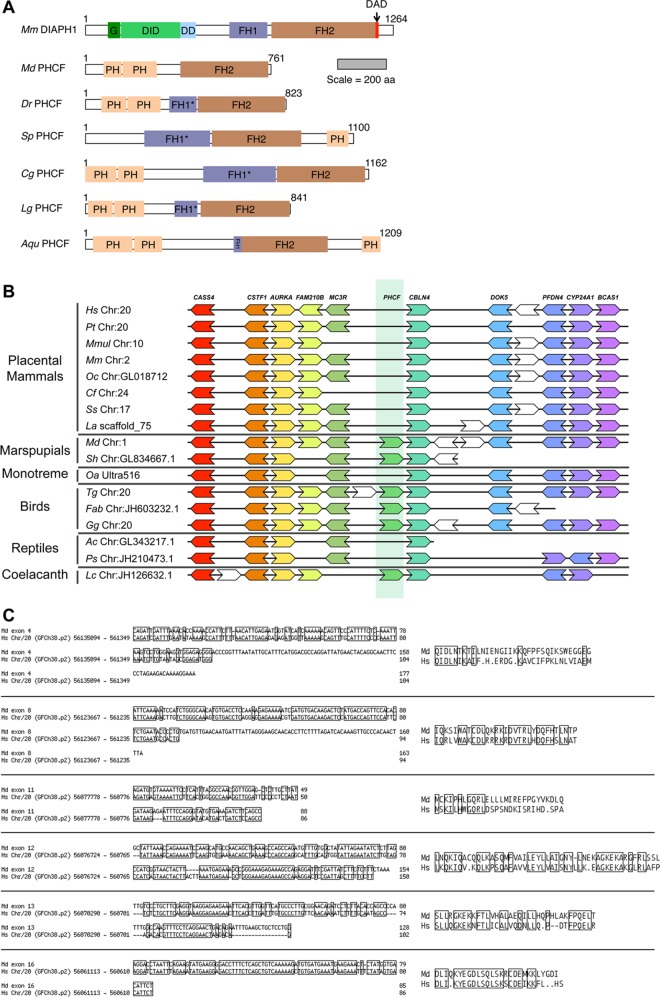
PH domain-containing formins (PHCFs) in vertebrates and other metazoans. **(A) Domain organizations of PHCF homologs.** Based on predicted structural domains, PH domain-containing formins are present in the opossum *M*. *domestica* (Md), zebrafish *D*. *rerio* (Dr), purple sea urchin *S*. *purpuratus* (Sp), Pacific oyster *C*. *gigas* (Cg), owl limpet *L*. *gigantea* (Lg), and sponge *A*. *queenslandica* (Aqu). The domain organization of the *M*. *musculus* (Mm) Drf-type DIAPH1 formin is shown for comparison. The scale bar indicates protein length in amino acid residue number. **(B) Conserved synteny in the chromosomal neighborhood of vertebrate PHCF genes.** The chromosomal neighborhood of the *MC3R* and *CBLN4* genes is shown for human *H*. *sapiens* (Hs), common chimpanzee *Pan troglodytes* (Pt), rhesus macaque *Macaca mulatta* (Mmul), mouse *M*. *musculus* (Mm), rabbit *Oryctolagus cuniculus* (Oc), dog *Canis familiaris* (Cf), pig *Sus scrofa* (Ss), elephant *Loxodonta africana* (La), opossum *M*. *domestica* (Md), Tasmanian devil *Sarcophilus harrisii* (Sh), platypus *Ornithorhynchus anatinus* (Oa), zebra finch *Taeniopygia guttat* (Tg), flycatcher *Ficedula ablicollis* (Fab), chicken *G*. *gallus* (Gg), anole lizard *Anolis carolinensis* (Ac), Chinese turtle *Pelodiscus sinesnsis* (Ps), and coelacanth *Latimeria chalumnae* (Lc). Gene order is largely conserved, but a PHCF-coding gene (medium green) is present in this region only in coelacanth, birds, and marsupials. Distances are not drawn to scale, and white genes have no homologs. **(C) Blocks of homology to the opossum PHCF coding sequence in the human genome.** (Left) Six blocks of sequence in the human genome between the *MC3R* and *CBLN4* genes can be aligned with parts of six predicted exons of the opossum PHCF-coding gene. (Right) Conceptual translations of the human sequences produce in-frame stop codons and shifts in reading frame relative to the opossum sequence, consistent with absence of a functional human PHCF-coding gene.

A study by Chalkia and colleagues [19] had identified a formin from *N*. *vectensis* that, based on FH2 domain sequence, was unrelated to any of the seven metazoan formin subtypes known at that time. This formin also differed from other metazoan formins in having N- and C-terminal PH domains. From the broader sampling of species here, additional PH domain-containing formins (PHCFs) were revealed in additional metazoans. Analysis of these proteins using the PHYRE^2^ website [33] predicted the PHCF of the sponge *A*. *queenslandica* also has N- and C-terminal PH domains, specifically a tandem pair in the N-terminus and a single C-terminal one (Fig 2A). Chordate and mollusk PHCFs were predicted to also encode a pair of N-terminal PH domains but lack a C-terminal one, whereas an echinoderm PHCF was predicted to have a C-terminal PH domain but none within its N-terminus (Fig 2A). The PHCFs showed no additional formin homology, except for proline-rich putative FH1 domains that showed some variability between homologs. Some PHCF FH1 domains appeared unremarkable, but in several homologs, extended stretches of non-proline residues interrupted their proline-rich regions, while the PHCF of the opossum *M*. *domestica* lacked any proline-rich region N-terminal to its FH2 domain (Fig 2A). Despite these differences, the FH2 domains of all PHCFs clustered together in phylogenetic trees (Fig 1 and S1 and S2 Figs), indicating a common origin for these proteins as an eighth conserved subtype of metazoan formin.

PHCFs are present in many vertebrates, including marsupial mammals, but are absent from placental mammals, suggesting they were lost from that lineage relatively recently. Examination of vertebrate genomes using the Genomicus database ([41]*) showed that PHCF-coding genes are positioned between the *MC3R* and *CBLN4* genes in vertebrates that range from coelacanths to birds to marsupials (Fig 2B). For the most part, synteny in this region is conserved in placental mammalian genomes, with the exception that there is no predicted formin-coding gene in this location (Fig 2B). To probe for evidence that an ancestral PHCF-coding gene might have once been present, the entire human genome was subject to a BLAST search using the predicted opossum PHCF cDNA. Six discrete stretches of sequence homology were identified that correspond to portions of six of seventeen predicted coding exons of the opossum PHCF gene (Fig 2C). Notably, all of these fell between *MC3R* and *CBLN4* in the human genome. However, BLAST searches could not identify expressed sequence tags from any placental mammal that were homologous to PHCF. Moreover, nonsense mutations in the human sequences are predicted to introduce in-frame stop codons, and insertions and deletions are predicted to result in shifts in reading frame (Fig 2C), all consistent with an ancestral PHCF formin gene that no longer produces a functional formin.

### A ninth formin subtype related to ecdysozoan MWH proteins

**Fig 3:**
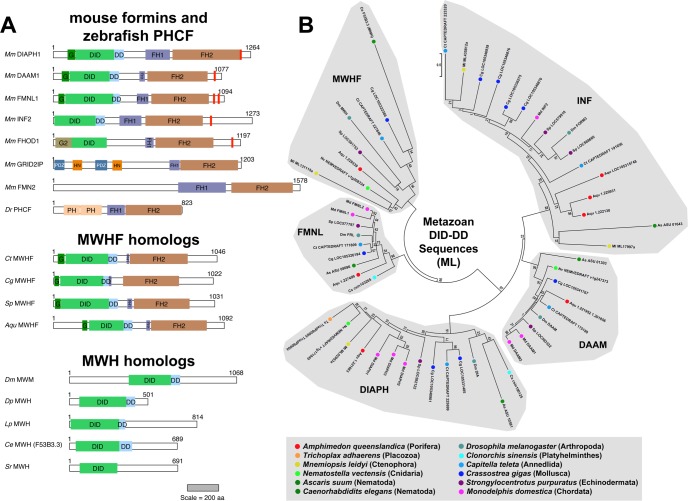
Conserved DID-DD sequences in formins and MWH proteins. **(A) Predicted domain organization of DID- and DD-containing metazoan proteins.** Mouse formins of the canonical subtypes shown for comparison are: Drf-type DIAPH1, DAAM1, and FMNL1; Drf-like INF2 with a comparatively truncated N-terminus; and non-Drf-type FHOD1 with a structurally distinct GTPase-binding domain (G2), GRID2IP with PDZ and Harmonin N-terminus-like (HN) domains, and FMN2 with a structurally divergent N-terminus. Also shown is the zebrafish non-Drf PHCF with N-terminal PH domains. The presence of C-terminal DAD/WH2-like motifs (red bars) generally correlates with the presence of an N-terminal DID. (Middle) Shown are predicted domain organizations for MWHF proteins identified in the polychaete worm *C*. *teleta* (Ct), the Pacific oyster *C*. *gigas* (Cg), the purple sea urchin *S*. *purpuratus* (Sp), and the sponge *A*. *queenslandica* (Aqu). Despite a Drf-type N-terminus, these proteins lack C-terminal DAD/WH2-like sequences. (Bottom) Drf-like DID and DD are also predicted for MWH of *D*. *melanogaster* (Dm), and related proteins in the water flea *D*. *pulex* (Dp), the horseshoe crab *L*. *polyphemus* (Lp), and the roundworms *C*. *elegans* (Ce) and *S*. *ratti* (Sr). Scale bar indicates protein lengths in amino acid residue number. **(B) ML phylogenetic tree of metazoan DID and DD sequences.** The evolutionary history for 56 DID-DD sequences from formins and MWH homologs was inferred by the ML method for 227 amino acid positions occupied in ≥ 90% of sequences. All bootstrap values are shown, and the scale bar indicates the number of substitutions per site for branch lengths. Proteins were selected for analysis from representatives of eleven phyla. Because no MWH-related protein could be detected in the representative nematode *A*. *suum*, the MWH-like protein F53B3.3 from *C*. *elegans* was also included in this analysis. For each formin, the subtype established based on FH2 domain (Fig 1) was recapitulated based on DID-DD. MWH DID-DD sequences grouped with a novel subtype of MWH-related formins (MWHFs). Note, ML trees generated without MWH proteins are otherwise essentially unchanged.

A ninth cluster of formins in each FH2 phylogenetic tree was linked with, but separated from, the FMNL proteins by well-supported nodes with bootstrap values > 85 in all trees (the MWHF group seen in Fig 1 and S1 and S2 Figs). These formins are predicted to be Drfs with a domain organization of G-DID-DD-FH1-FH2 (Fig 3A). As a further test of whether these formins constitute a distinct subtype, their N-terminal DID-DD sequences were aligned with those of other Drfs (S1 Text, Alignment 3), and ML and NJ phylogenetic trees were estimated (Fig 3B and S3 Fig). Again, this group of proteins formed a strongly supported distinct subtype positioned adjacent to the FMNL proteins.

The product of the *multiple wing hairs* gene of *D*. *melanogaster* was shown to also have homology to Drf-type formin Interpro GTPase-binding domain (DrfGBD) and formin homology-3 (FH3) domain [23, 24]. In terms of structural domains, the Interpro DrfGBD corresponds to a G-domain and a portion of a DID, while the FH3 domain corresponds to the remainder of a DID plus a DD [45]. Analysis of *D*. *melanogaster* MWH protein using the PHYRE^2^ website [33] predicted the presence of a DID and DD, but no G-domain (Fig 3A). Sensitive BLAST searches identified MWH homologs in other insects, non-insect arthropods, and a subset of nematodes (S1 Table), and these were also predicted to adopt DID-DD folds (Fig 3A).

**Fig 4:**
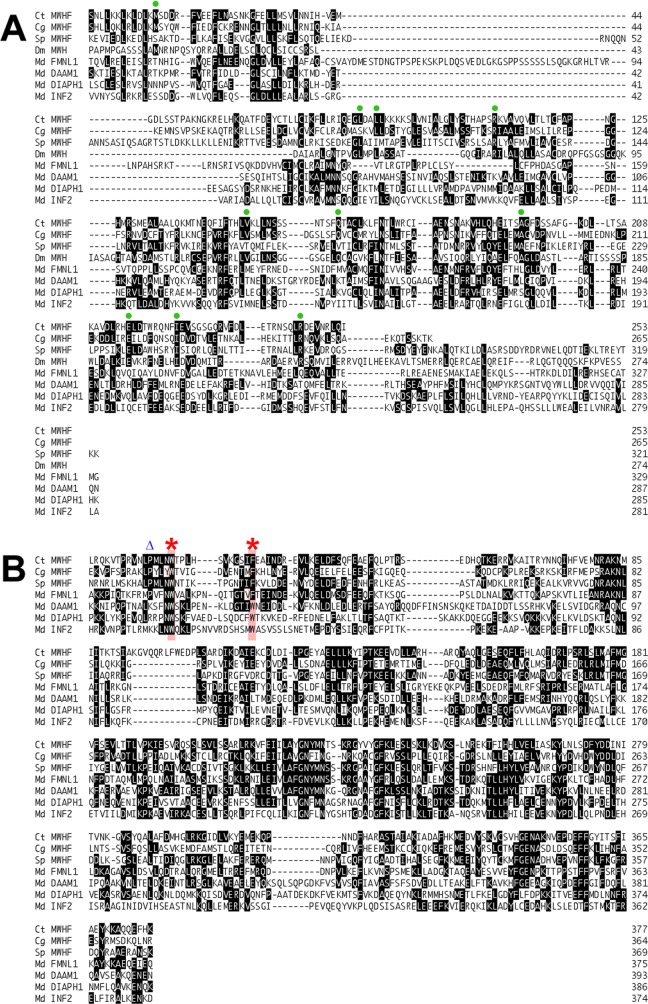
Sequence similarities between MWHF, MWH, and FMNL proteins. **(A) DID and DD sequences.** Shown are a subset of amino acid sequences used to estimate the DID-DD phylogenetic tree of Fig 3B, including those from the MWHF proteins of the polychaete worm *C*. *teleta* (Ct), the Pacific oyster *C*. *gigas* (Cg), and the purple sea urchin *S*. *purpuratus* (Sp), MWH from *D*. *melanogaster* (Dm), and the formins FMNL1, DAAM1, DIAPH1, and INF2 from the opossum *M*. *domestica* (Md). Green circles indicate amino acid positions for which MWH and two or more MWHFs are identical but distinct from other formins. **(B) FH2 domain sequences**. Shown are sequences from the same subset of formins used to estimate the FH2 domain phylogenetic tree of Fig 1. Red asterisks indicate conserved aromatic residues of the FH2 domain lasso. The second aromatic residue is phenylalanine in FMNL and MWHF proteins, but tryptophan in all other formins. A blue triangle indicates a position uniquely occupied by proline in FMNL and MWHF proteins.

The DID-DD sequences of *D*. *melanogaster* MWH and *C*. *elegans* MWH-related F53B3.3 were also included in ML and NJ phylogenetic trees estimated using Drf-formin DID-DD sequences. Consistent with an earlier effort that was unable to assign MWH to a particular formin subtype [25], neither protein clustered with one of the four previously known Drf subtypes. Instead, both fell into the novel formin subtype positioned close to the FMNL proteins (Fig 3B and S3 Fig). For this reason, this novel subtype is designated here as the MWH-related formins (MWHFs). Consistent with this, inspection of aligned DID-DD sequences shows many regions of similarity shared between MWH and MWHF proteins, but not other Drfs (Fig 4A, *green circles*).

The close position of FMNL and MWHF proteins on phylogenetic trees implies a particular relatedness between these two groups of formins. Casual inspection of aligned DID-DD and FH2 sequences (Fig 4) reveals only a very modest increased similarity between MWHF and FMNL proteins relative to other Drf-type formins. However, FMNL and MWHF subtypes do share two unique sequence features in the 'lasso' region of their FH2 domains. A conserved feature of the lasso for all formins is a pair of aromatic residues (Fig 4B, *red asterisks*). All other formins encode tryptophan at these positions, but the FMNL and MWHF proteins substitute phenylalanine for the second tryptophan. Less striking, but also unique for the FMNL and MWHF homologs, is the presence of proline at the fourth residue position upstream of the first conserved tryptophan (Fig 4B, *blue triangle*).

A distinctive feature of MWHF proteins compared to most other Drf-type formins is that they lack any detectable DAD- or WH2-like motifs C-terminal to their FH2 domains (Fig 3A). This is particularly surprising when considering that FMNL proteins generally have two C-terminal DAD/WH2 motifs [12, 14].

### Nematode formins and FOZI-1-related proteins

Some nematode FH2 domains are particularly divergent, and consequently several previous studies were unable to assign subtypes to some *C*. *elegans* proteins [17–19, 22]. In this analysis, all FH2 domains from the nematode *A*. *suum* fell into one of five subtypes (DIAPH, DAAM, FMNL, FHOD, or INF) (Fig 1 and S1 Fig). When FH2 domain sequences from four additional roundworms (including *C*. *elegans*) were examined as part of a larger array of bilaterian species, most of these also grouped with one of these five subtypes (S2 Fig), with a few exceptions discussed below. Further supporting these subtype assignments, DID-DD sequences of the *A*. *suum* DIAPH, DAAM, and FMNL homologs, and one of its INF homologs, and the DID sequence of its FHOD homolog, all clustered with formins of the appropriate subtype in ML or NJ phylogenetic trees (Fig 3B and S4 Fig). This matched previous results for conserved N-terminal sequences of the *C*. *elegans* formins [46].

**Fig 5:**
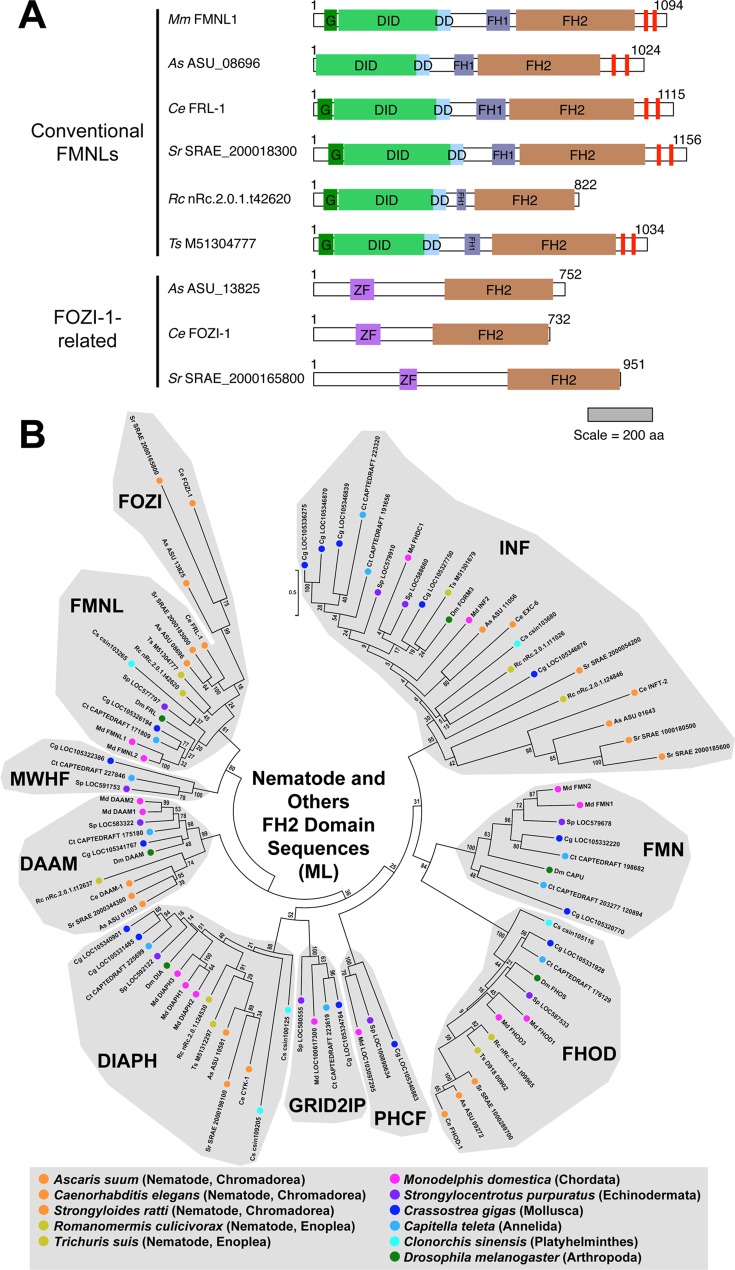
Divergent FH2 domains of nematode FOZI-1-like proteins belong to the FMNL subtype. **(A) Domain organizations of conventional nematode FMNL homologs and FOZI-1-related proteins.** Predicted structural domains are shown for FMNL-subtype FH2 domain-containing proteins from *M*. *musculus* (Mm) and five nematodes, *A*. *suum* (As), *C*. *elegans* (Ce), *S*. *ratti* (Sr), *R*. *culicivorax* (Rc), and *T*. *suis* (Ts). The nematode proteins fall into two classes. The conventional FMNL proteins largely resemble *M*. *musculus* FMNL1 with a Drf-type N-terminal domain organization (G-DID-DD) and pair of WH2/DAD-like motifs (red bars) C-terminal to the FH2 domain. The second set of proteins, including *C*. *elegans* FOZI-1, have N-terminal zinc fingers (ZF) followed by a C-terminal FH2 domain and no additional formin homology. The scale bar indicates protein length in amino acid residue number. **(B) ML phylogenetic tree of full-length nematode and other bilaterian FH2 domain sequences.** The evolutionary history for 93 FH2 domain sequences from five nematodes belonging to two orders (chromadorea and enoplea), and from six non-nematode bilaterians, was inferred by the ML method for 280 amino acid positions fully occupied in all sequences. All bootstrap values are indicated, and the scale bar indicates the number of substitutions per site for branch lengths. All nematode FH2 domains fell within one of the nine subtypes. Within the FMNL subtype, the divergent FOZI-1-related proteins of the chromadorean nematodes formed a subgroup that was positioned closely to the chromadorean conventional FMNL formins.

The notable exception to these straightforward assignments was a set of FH2 domain-containing proteins that included the highly divergent FOZI-1 of *C*. *elegans*. Based on analysis using the PHYRE^2^ database, these proteins lack any formin homology outside their FH2 domain, but encode two N-terminal zinc fingers (Fig 5A). In the FH2 domain phylogenetic tree estimated using the larger number of bilaterian formins, the FOZI-1-like proteins segregated from all other subtypes (S2 Fig). However, it seemed unlikely that these proteins represent a novel subtype found in no other metazoan. Rather, it seemed more likely that their segregation from other formins was an artifact of estimating a phylogenetic tree using partial sequences. That is, using partial sequences necessitated inclusion in the analysis of residue positions for which some sequences had gaps. FOZI-1-like FH2 domains are distinct in that they are truncated in the highly conserved "knob" region [20, 21], and consideration of these vacated positions may reinforce an apparent divergence of these proteins. To attempt to avoid this, the nematode FH2 domain sequences were aligned with a set that included only full-length FH2 domain sequences (S1 Text, Alignment 4). When a new ML phylogenetic tree was estimated using only fully occupied positions, the FOZI-1-like FH2 domains clustered within the FMNL subtype (Fig 5B). Consistent with this assignment, FOZI-1-like FH2 domains also substitute phenylalanine for tryptophan as the second conserved aromatic residue of the lasso region.

A survey of sequenced nematode genomes through the WormBase ParaSite webpage (parasite.wormbase.org; version WBPS6) [29] revealed FOZI-1 homologs are present in nematodes of the order chromadorea, but not of the order enoplea (examples shown in Fig 5A). Within the FMNL subtype in the FH2 domain phylogenetic tree (Fig 5B), nematode proteins formed three distinct subgroups: a modestly supported (bootstrap value 45) group of enoplean conventional FMNL proteins, a very strongly supported (bootstrap value 100) group of chromadorean conventional FMNL proteins, and a very strongly supported (bootstrap value 99) group of chromadorean FOZI-1-like proteins (Fig 5B). Interestingly, the chromadorean FOZI-1-like subgroup was more closely associated with the chromadorean conventional FMNL group than to other proteins. Although this was only modestly supported (bootstrap value 24), it suggests FOZI-1-related proteins are most closely related to their conventional chromadorean counterparts. A possible explanation for this is that FOZI-1-type proteins arose from a duplication of an ancestral FMNL-coding gene in chromadorea after its divergence from enoplea. A subsequent fusion of one of the FH2 domain-coding sequences with a zinc finger-coding sequence would have produced the FOZI-1-type proteins.

## Discussion

The purpose of this study was to address lingering questions about the relatedness of a handful of formin and formin-related proteins, particularly nematode formins, MWH, and a PH domain-containing formin of cnidaria. To improve upon earlier studies, this analysis included formins from metazoan phyla not previously analyzed (porifera, placozoa, ctenophora, and platyhelminthes), and from additional species from phyla only rarely analyzed in other studies (mollusca and annelida). Two major findings were discovery that the metazoan formin family is more diverse than previously appreciated, and that all formins and formin-related proteins are members of evolutionarily conserved subtypes that were likely present at the very origins of metazoa.

**Fig 6:**
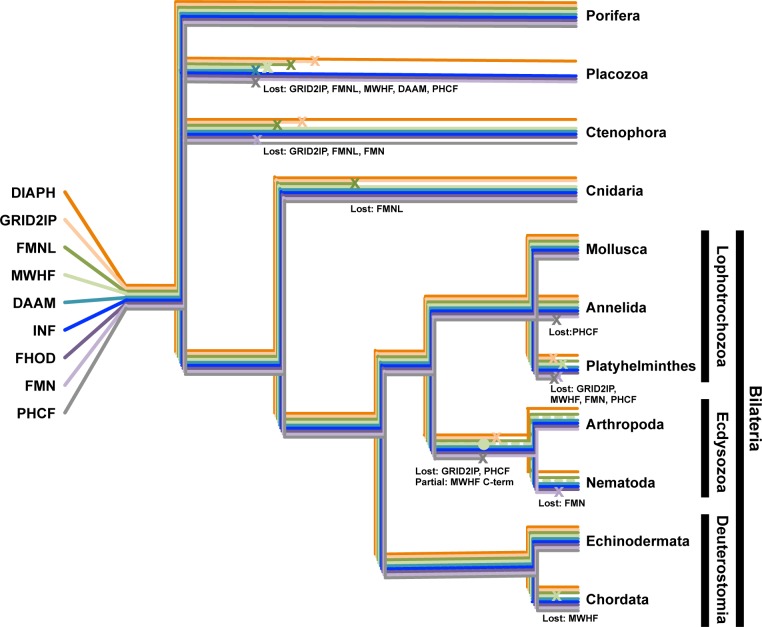
Patterns of formin subtype losses in metazoa. The current disposition of formin subtypes among the metazoan phyla can be explained by the presence of all nine subtypes in the last common metazoan ancestor, and subsequent subtype losses (x) or partial losses (•) in different lineages. Because the evolutionary relationships among basal phyla (porifera, placozoa, and ctenophora) remain under debate [42–44], those phyla have each been drawn as independent branches. Five formin subtypes have been lost from placozoa (GRID2IP, FMNL, MWHF, DAAM, and PHCF), three from ctenophora (GRID2IP, FMNL, and FMN), and one from cnidaria (FMNL). Among the lophotrochozoans, one subtype has been lost from annelida (PHCF), and four from platyhelminthes (GRID2IP, MWHF, FMNL, and PHCF). Note, no helminth DAAM subtypes were displayed in phylogenetic trees, but the planaria *Schmidtea mediterranea*, which was not included in estimating those trees, encodes a DAAM-related formin. In the common ecdysozoan ancestor to arthropods and nematodes, two subtypes were lost (GRID2IP and PHCF), and the C-terminus of the MWHF subtype was lost, resulting in MWH proteins. In nematodes, an additional loss of the FMN subtype occurred. Among the deuterostomes, chordates lost a single subtype (MWHF). Porifera, mollusca and echinodermata retain homologs of all nine subtypes. Events along the same braches were positioned for visual clarity, and are not meant to imply relative timing.

This analysis revealed nine formin subtypes, each with broad representation across the animal phyla (Fig 6). These included the seven subtypes well known from earlier studies—DAAM, DIAPH, FHOD, FMN, FMNL, INF, and GRID2IP/delphilin [17, 18]—as well as two novel subtypes (Fig 1). One of these novel subtypes is characterized by N- and/or C-terminal PH domains (Fig 2A). This represents an expansion of a PH domain-containing formin (PHCF) subtype previously known only from a single representative each from the cnidarian *N*. *vectensis* and the non-metazoan choanoflagellate *Monosiga brevicollis* [19]. The second novel subtype is called here MWH-related formins (MWHFs) for their relatedness to portions of the *D*. *melanogaster* MWH protein (Fig 3B and see below). The existences of all nine subtypes were strongly supported by nodes with high bootstrap values in phylogenetic trees estimated from FH2 domain sequences and, when possible, DID-DD sequences (Figs 1 and 3B).

Considering the extensive focus the formin family has received over the past decade, it was surprising to discover PHCF and MWHF proteins as two overlooked formin groups. However, this is readily explained by absence of these formin subtypes from the animals most commonly studied in phylogenetic analyses: placental mammals, insects, and nematodes. In the case of the MWHF proteins, their similarity to the FMNL proteins also contributed to their previous obscurity, with some MWHF proteins having been mistakenly categorized as FMNLs in past studies [19, 46]. However, the FMNL and MWHF subtypes are readily resolved when analyzing formins from multiple species that encode homologs of both subtypes.

MWHFs are present in a broad range of phyla, including several basal metazoan branches, as well as in the bilaterian phyla echinodermata, mollusca, and anellida. Their name derives from their relatedness to the MWH protein of *D*. *melanogaster*. That is, MWH is predicted to have Drf-related DID and DD, but further formin homology [23, 24]. In phylogenetic trees estimated for DID and DD sequences, MWH clusters with this novel formin group (Figs 3 and 4A). The presence of additional MWH homologs in other arthropods and also in some nematodes (Fig 3A) suggests that their common ecdysozoan ancestor also encoded a MWHF subtype formin, whose C-terminus was lost to produce the MWH proteins (Fig 6).

MWHFs are positioned in phylogenetic trees close to the FMNL formins (Figs 1 and 3B), and they share several unique sequence features in the lasso region of their FH2 domains (Fig 4B). The flexible lasso plays a critical role in dimerization of FH2 domains by enwrapping the 'post' region of an opposing FH2 domain [47]. As part of this interaction, two highly conserved aromatic side chains of the lasso embed into hydrophobic pockets of the post. These two residues are tryptophans in every formin examined here, except in all the FMNLs and MWHFs, for which phenylalanine is substituted for the second aromatic residue (Fig 4B). The functional significance of this difference remains to be determined.

One MWHF feature distinct from other Drf-type formins is the apparent absence of DAD or WH2-like motifs from their C-terminus (Fig 3A). These motifs have been shown in many cases to interact with actin monomers, actin filaments, or both, and in different formins, enhance actin filament nucleation, bundling, or severing, or processivity of the formin at the elongating barbed end [11–14, 48]. These motifs are usually present among metazoan formins that have an N-terminal DID (Fig 3A), and in many cases, the DID and DAD/WH2 interact. Frequently, though not always, this interaction has an autoinhibitory effect [15]. One implication of absence of DAD/WH2 motifs is that MWHFs might not be subject to autoinhibition. However, these motifs are very poorly conserved, and cryptic ones might have been missed here. Also, studies of the *D*. *melanogaster* FMN formin, CAPU, provide a cautionary tale against assumptions based on sequence identity. The non-Drf CAPU lacks DID or DAD homology, but its N- and C-termini still interact in an autoinhibitory manner [49]. Moreover, the CAPU C-terminal tail enhances processivity, similar to the effects of some DAD/WH2-containing formin tails [48]. Thus, it is important to directly test whether MWHF C-termini have similar effects.

PHCF proteins are also broadly represented across the animals, appearing in the phyla porifera, ctenophora, cnidaria, mollusca, echinodermata, and chordata (Fig 1). PHCFs were not identified in any placental mammal, but their presence in such vertebrates as fish, birds, and even marsupial mammals, suggests that their loss was a relatively recent event. Consistent with this, the human chromosomal locus corresponding to the location of the PHCF-coding gene in other vertebrates has stretches of homology to PHCF-coding sequence (Fig 2B and 2C). However, the apparent absence of expressed sequences from this locus in any placental mammal, and the presence of mutations predicted to introduce premature stops and shifts in reading frame of the human sequences, all suggest that no functional PCHF is produced in humans.

The distribution of formin subtypes across the animal kingdom, and particularly their presence among basal phyla, suggests all nine subtypes were already present in the last common metazoan ancestor, and that most phyla subsequently lost one or more subtypes (Fig 6). Interestingly, all species examined here encode at least one DIAPH and INF homolog, while other isoforms were missing from one or more species (S1 Table), suggesting DIAPH and INF proteins might play roles critical to animal biology. While various functions have been described for INF proteins in different species [5, 6, 50, 51], it remains unclear if there is an evolutionarily conserved function that would have driven preservation of the INFs across the animals. However, the DIAPH formins have been tied to cytokinetic contractile actin ring assembly in a variety of animal systems [52–54], a function that could easily explain the universal retention of this subtype. Conversely, in cases where formins have been lost from particular groups of organisms, their roles had presumably become dispensable.

## Conclusions

The increasing availability of annotated genomic data allows us to use phylogenetic analyses to continually sharpen our views of relationships between proteins of diverse species. For a family like the formins, where many model systems are employed, this is important, allowing us to appreciate which proteins are true homologs, and which are not. For example, we confirm that nematode formins, including those of the model organism *C*. *elegans*, are homologs of conserved subtypes. Perhaps more importantly, revised analyses sometimes result in humbling realizations about how much might remain to be learned. For example, it was startling to discover that many of our closest cousins bear a PH domain-containing formin, and that we ourselves have a detectable genomic scar of its former presence. It seems very likely that this will not be a final catalog of the animal formin subtypes.

## Supporting Information

S1 Table List of formins used in this study. (XLSX)

S1 Text Multiple sequence alignments in interleaved format. (TXT)

S1 Fig NJ phylogenetic tree of metazoan FH2 domains. The evolutionary history for 100 FH2 domain amino acid sequences from representatives of eleven metazoan phyla was inferred by the NJ method for 343 amino acid positions occupied in ≥ 95% of sequences. All bootstrap values are indicated, and the scale bar indicates the number of substitutions per site for branch lengths. As found in the corresponding ML tree (Fig 1), nine groups populated by formins from multiple species clustered behind nodes with bootstrap values ≥ 50, suggesting the presence of nine evolutionarily conserved subtypes. Seven of these conformed to the previously recognized DAAM, DIAPH, FHOD, FMN, FMNL, INF and GRID2IP subtypes, while two others, designated MWHF and PHCF, were novel. Asterisks (*) indicate formins for which a partial FH2 domain sequence was used for this analysis.
(TIF)

S2 Fig ML phylogenetic tree of FH2 domains from twenty-one bilaterian species. The evolutionary history for 227 FH2 domain amino acid sequences was inferred by the ML method for 295 amino acid positions occupied in ≥ 95% of sequences. All bootstrap values are indicated, and the scale bar indicates the number of substitutions per site for branch lengths. Asterisks (*) indicate formins for which a partial FH2 domain sequence was used for this analysis. All formins, with the exception of three nematode proteins, fell into one of nine conserved subtypes.
(TIF)

S3 Fig NJ phylogenetic tree of metazoan DID-DD sequences. The evolutionary history for 56 DID-DD sequences of metazoan Drf-type formins and MWH homologs representing eleven metazoan phyla was inferred by the NJ method for 227 amino acid positions occupied in ≥ 90% of sequences. All bootstrap values are shown, and the scale bar indicates the number of substitutions per site for branch lengths. Because the representative nematode *A*. *suum* lacked a detectable MWH-related protein, the DID-DD of the *C*. *elegans* MWH-related F53B3.3 was included in this analysis. Results shown here match those of the corresponding ML phylogenetic tree (Fig 3B), including the grouping of MWH homologs with MWHF proteins. Note, an NJ tree generated without MWH proteins is otherwise essentially unchanged.
(TIF)

S4 Fig Phylogenetic trees of metazoan DID sequences. DID sequences from Drf-type DIAPH, DAAM, FMNL, MWHF, and INF formins, and non-Drf-type FHOD formins from the indicated species were identified and aligned (S1 Text, Alignment 5). **(A) NJ phylogenetic tree of DIDs.** The evolutionary history for 48 DIDs was inferred by the NJ method for 164 amino acid positions occupied in ≥ 90% of sequences. All bootstrap values are shown, and the scale bar indicates the number of substitutions per site for branch lengths. Based on DID sequences, formins segregated into the same subtypes as observed after analysis of their FH2 domain sequences (Fig 1). **(B) ML phylogenetic tree of DIDs.** The evolutionary history of the same sequences was also inferred by the ML method. Similar to the NJ tree, most formins segregated into the same subtypes. The exception was a disruption of the DIAPH subtype by the internal placement of the branch leading to the FHOD subtype formins.
(TIF)
