# Supplementary figures and images for: Revisiting the Phylogeny of the Animal Formins: Two New Subtypes, Relationships with Multiple Wing Hairs Proteins, and a Lost Human Formin
Source: PLoS One. 2016 Oct 3;11(10):e0164067. doi: 10.1371/journal.pone.0164067 (PMC5047451; doi:10.1371/journal.pone.0164067)

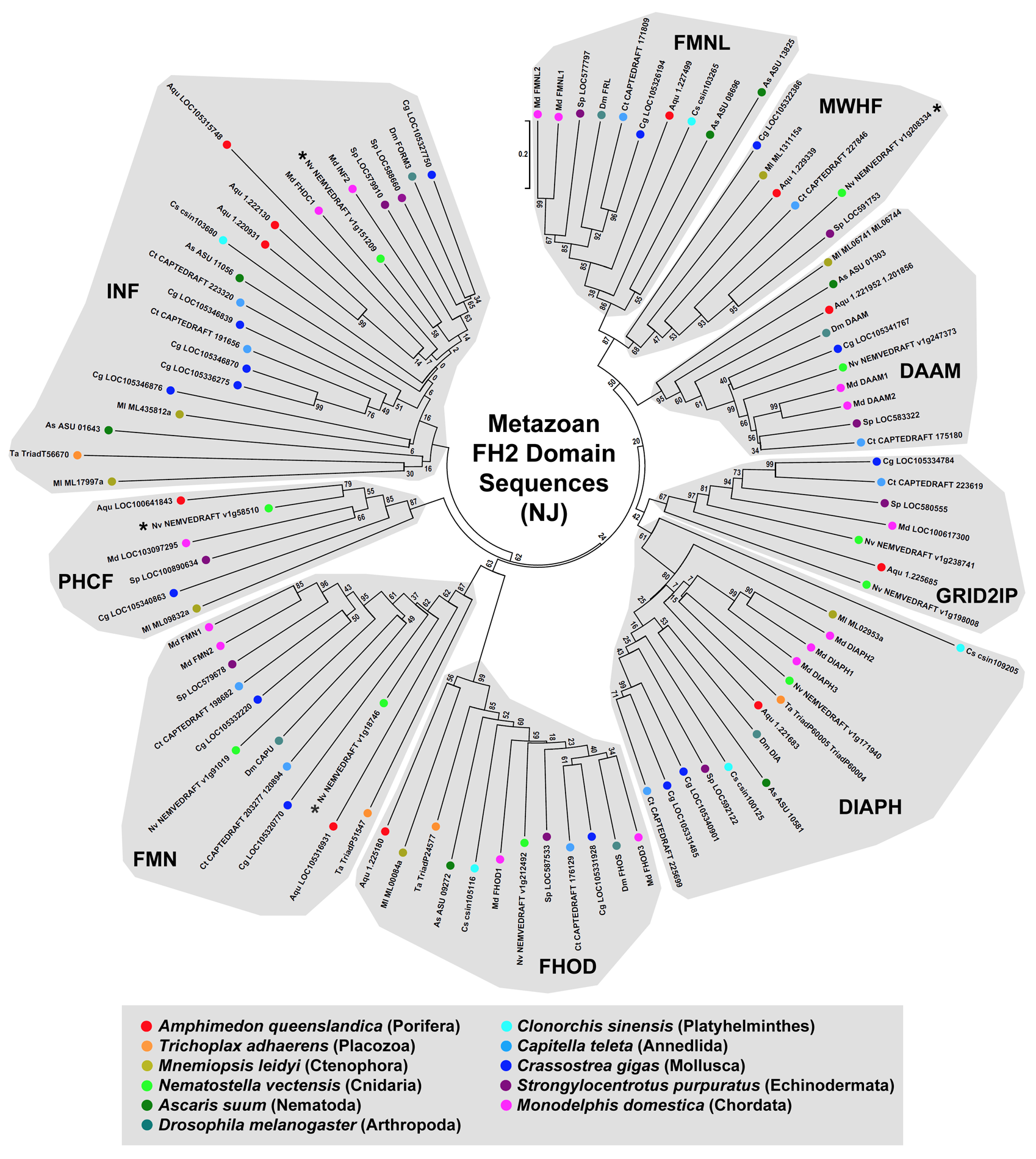

Supplement: S1 Fig — The evolutionary history for 100 FH2 domain amino acid sequences from representatives of eleven metazoan phyla was inferred by the NJ method for 343 amino acid positions occupied in ≥ 95% of sequences. All bootstrap values are indicated, and the scale bar indicates the number of substitutions per site for branch lengths. As found in the corresponding ML tree (Fig 1), nine groups populated by formins from multiple species clustered behind nodes with bootstrap values ≥ 50, suggesting the presence of nine evolutionarily conserved subtypes. Seven of these conformed to the previously recognized DAAM, DIAPH, FHOD, FMN, FMNL, INF and GRID2IP subtypes, while two others, designated MWHF and PHCF, were novel. Asterisks (*) indicate formins for which a partial FH2 domain sequence was used for this analysis. (TIF) [file pone.0164067.s003.tif]

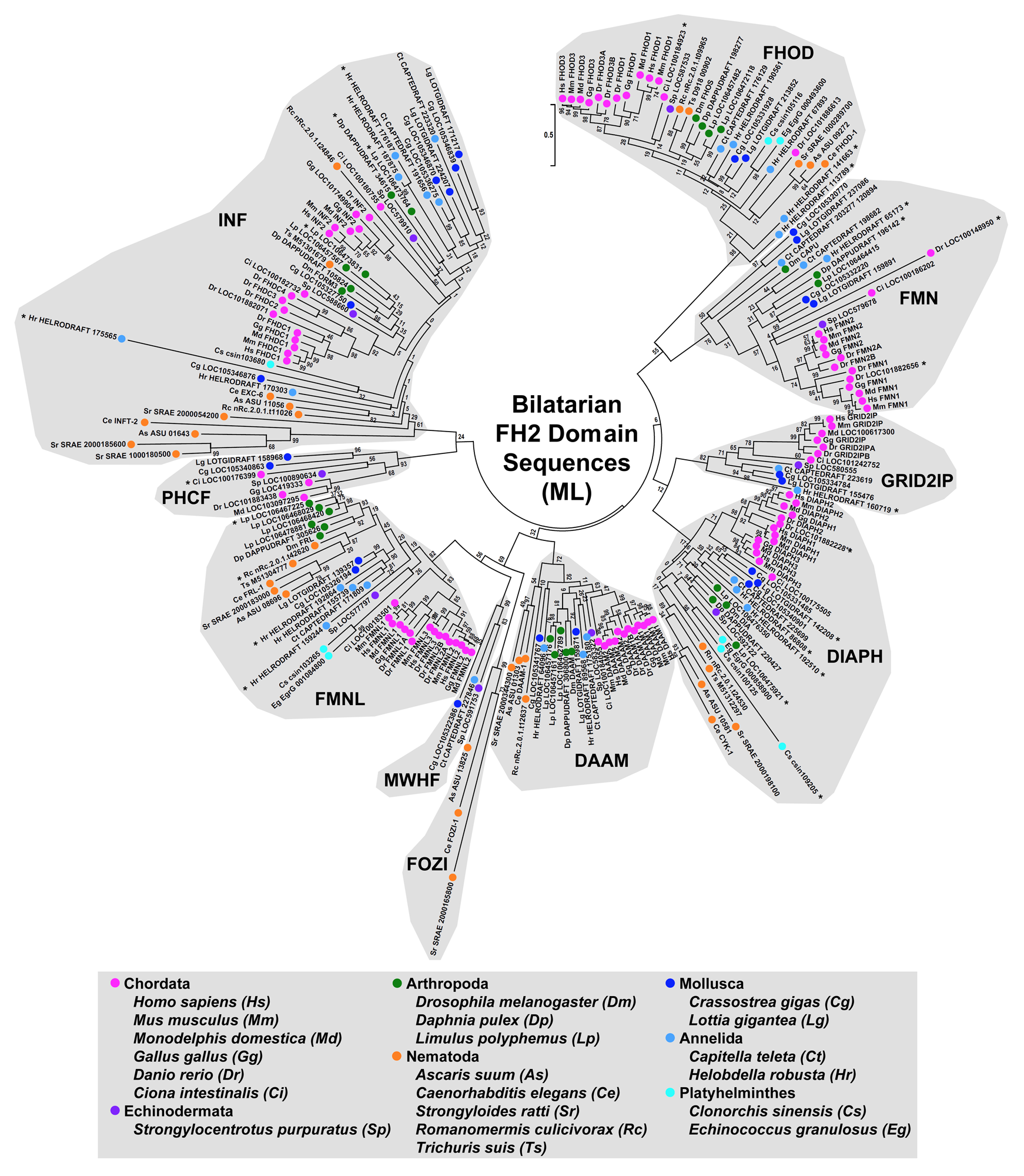

Supplement: S2 Fig — The evolutionary history for 227 FH2 domain amino acid sequences was inferred by the ML method for 295 amino acid positions occupied in ≥ 95% of sequences. All bootstrap values are indicated, and the scale bar indicates the number of substitutions per site for branch lengths. Asterisks (*) indicate formins for which a partial FH2 domain sequence was used for this analysis. All formins, with the exception of three nematode proteins, fell into one of nine conserved subtypes. (TIF) [file pone.0164067.s004.tif]

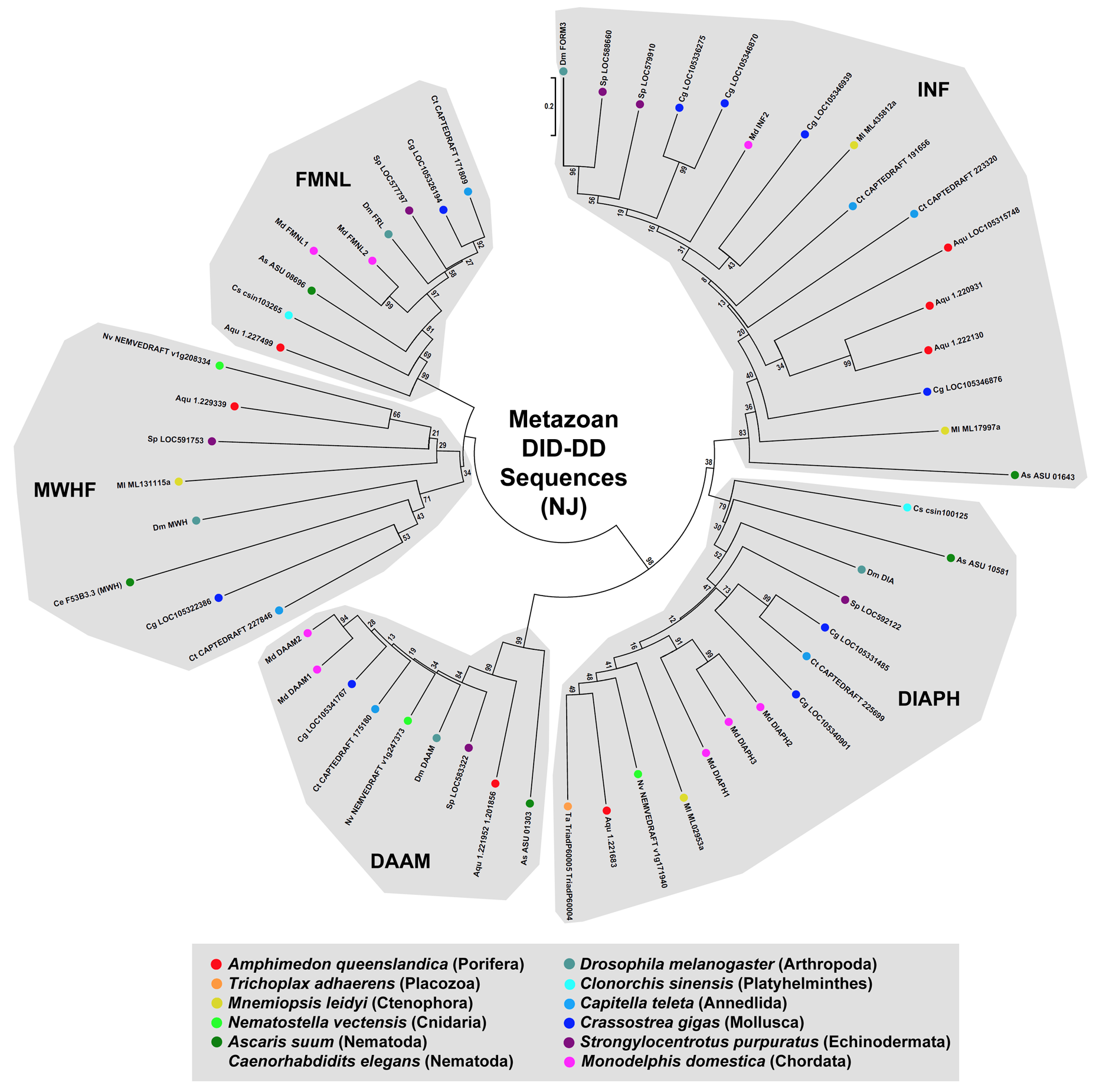

Supplement: S3 Fig — The evolutionary history for 56 DID-DD sequences of metazoan Drf-type formins and MWH homologs representing eleven metazoan phyla was inferred by the NJ method for 227 amino acid positions occupied in ≥ 90% of sequences. All bootstrap values are shown, and the scale bar indicates the number of substitutions per site for branch lengths. Because the representative nematode A. suum lacked a detectable MWH-related protein, the DID-DD of the C. elegans MWH-related F53B3.3 was included in this analysis. Results shown here match those of the corresponding ML phylogenetic tree (Fig 3B), including the grouping of MWH homologs with MWHF proteins. Note, an NJ tree generated without MWH proteins is otherwise essentially unchanged. (TIF) [file pone.0164067.s005.tif]

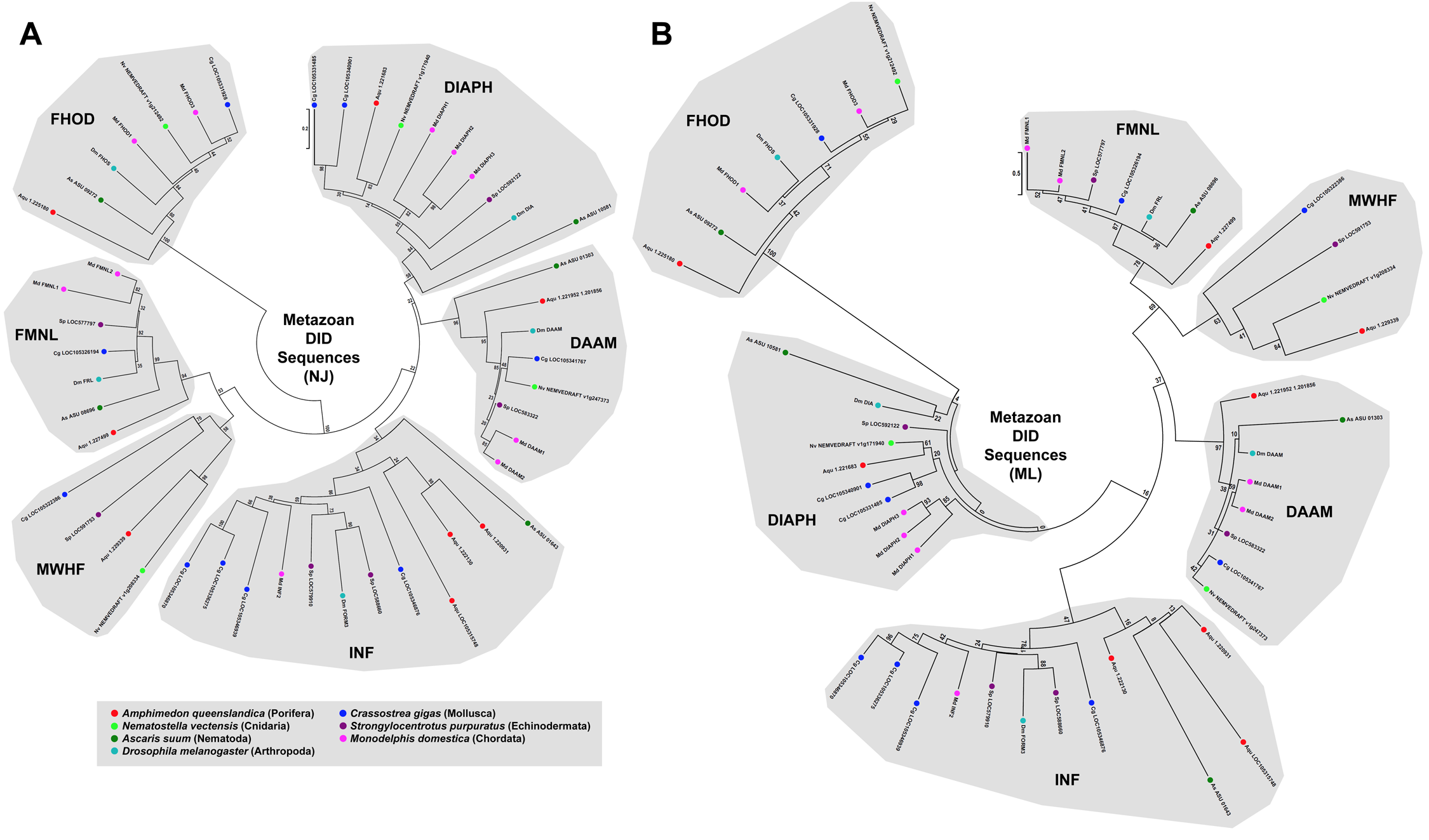

Supplement: S4 Fig — DID sequences from Drf-type DIAPH, DAAM, FMNL, MWHF, and INF formins, and non-Drf-type FHOD formins from the indicated species were identified and aligned (S1 Text, Alignment 5). (A) NJ phylogenetic tree of DIDs. The evolutionary history for 48 DIDs was inferred by the NJ method for 164 amino acid positions occupied in ≥ 90% of sequences. All bootstrap values are shown, and the scale bar indicates the number of substitutions per site for branch lengths. Based on DID sequences, formins segregated into the same subtypes as observed after analysis of their FH2 domain sequences (Fig 1). (B) ML phylogenetic tree of DIDs. The evolutionary history of the same sequences was also inferred by the ML method. Similar to the NJ tree, most formins segregated into the same subtypes. The exception was a disruption of the DIAPH subtype by the internal placement of the branch leading to the FHOD subtype formins. (TIF) [file pone.0164067.s006.tif]
